# An archetypal model of a breathable air-circuit in an electro-pneumatic ventilator device

**DOI:** 10.1016/j.heliyon.2022.e09378

**Published:** 2022-05-02

**Authors:** Ebenezer Olubunmi Ige, Adedotun Adetunla, Samuel Olufemi Amudipe, Adeyinka Adeoye, Matthew Glucksberg

**Affiliations:** a Department of Biomedical Engineering, Afe Babalola University, Ado-Ekiti, Nigeria; b Department of Mechanical and Mechatronics Engineering, Afe Babalola University, Ado-Ekiti, Nigeria; c Department of Biomedical Engineering, Northwestern University, Illinois, USA

**Keywords:** Electro-pneumatic, Mechanical ventilator, On-delay timer, Solenoid valve, Tidal volume

## Abstract

Mechanical ventilator is a machine that is mechanically designed to deliver breathable air in and out of the lungs to provide a breathing mechanism for a patient who is physically unable to breathe, it is an indispensable life-support device in critical care medicine and medical emergencies such as scenarios during the COVID-19 pandemic. This research presents a model design of the pneumatic circuit that is electronically controlled, by using computer-aided pneumatic rig over selected 5/3, 5/2, 3/2 solenoid gating valves, the performance of these valves must be investigated to ascertain the most appropriate valve to be used for the electro-pneumatic mechanical ventilator. An elaborate parametric investigation reported for volume-controlled ventilators illustrate the influences of key parameters on the dynamics of the ventilated respiratory system. This study presents the linearity of tidal volume, peak pressure and lung compliance for the parameters considered. However, the maximum pressure of the ventilation device increases slowly when the tidal volumes exceed 600 ml. In addition, influence of evacuation time of the ventilator predicted over high throughput in time regimes of 1 s; 1.2 s; 1.4 s; 1.6 s, and 1.8 s showed that the pressure platform in the pipe might not appear if the exhaust time of the ventilator is less than 1.6 s. The 5/2 solenoid valve was considered the best with consistent flowrate. The archetypal model of the pneumatic circuit developed in this research could find vital application in the design of patient-interfacing devices particularly in ventilators and neonatal incubator.

## 1 Introduction

The recent unprecedented heat of the novel coronavirus (nCOV-19 or SARS Cov-2) pandemic that affected the world has showed the importance of Mechanical Ventilators (MV), hospitals in many parts of advanced World was described as medical war zone because of the rate of hospitalization and critical need of ventilator to support coronavirus patients [1, 2]. The shortage of ventilators particularly in USA mandated the use of executive order known as Defense Production Act to enforce the production of specialized ventilators for American hospitals as means to curtail the escalating mortality. The intervention of ventilators in hospitals was seen to improve medical outcome in middle aged patients and patients with no history of underlining health difficulties. For instance, a US-based report revealed survival rate of 67.4% for ventilator-bound patients of coronavirus mostly below 70 years of age [3]. A similar observational study from hospitals across Germany showed 56% discharge rate among patients requiring mechanical ventilation [4]. To a large extent, mortality could be reduced using mechanical ventilators. Beyond the pandemic, the need for supported ventilation spans a broad spectrum of applications in clinical environment such as neonatal care, anesthesia and in surgical procedures for respiratory dysfunctions.

The incidences respiratory-related diseases pose the challenge to improve on the strategies for precision delivery of breathable air in ventilator for critical healthcare. Traditional method of operation of mechanical ventilators requires predefined settings based on pulmonological assessment of the patients, which may precipitate incidences of patient-ventilator asynchrony specifically during the early period of novel coronavirus (COVID-19) pandemic [5, 6, 7]. The procedure is usually within the description, which follows the analogy of an open-loop systems, for which real-time feedback from patient is being ignored which could result to ventilator-induced complications. In addition, open-loop control of mechanical ventilators lead to a precursor for high tidal that may exacerbate the conditions of patients with pre-existing health conditions [8, 9]. Feedback enabled control architecture with feedback, such as proportional assist ventilation (PAV), that produces inspiratory pressure at instantaneous rate in a manner that is proportional to the real-time demand of the patient condition [10, 11, 12]. It has been posited in established literatures that Neurally-adjusted Ventilated Assist Strategies (NAVAS) could perform autonomous control of breathable parameters thereby improving synchronization in the performance of the patient-interfacing device [13]. The PAV/NAVAS-type device could improve outcome of clinical ventilation in cases of obstructive pulmonary diseases, anesthesia and pandemic scenarios by reducing tendencies of respiratory muscle dysfunction and the possibility of success level in the weaning process.

Development in the field of computer science has impacted on the present progress in design medical devices such clinical ventilators. In the early 1990s, microcontrollers have been identified as vital in the construction of bellows-type ventilator to simulated respiratory flow pattern for instantaneous breathing [14]. In the 90s, some authors demonstrated the performance of computer-supported ventilator on veterinary animals. Computer-aided ventilators presented as alternative to mechanical bellow, computer-enabled bellow type devices to simulate breathing ventilation [15, 16]. Over the years, advancement in ventilator design has become computer-driven, weaning of patients utilizing ventilator as life support device is a critical process that leverages on computer-interface of ventilators because data-driven precision is required [17]. Strickland et al 1991 developed ventilator-bound expert system that could manage decisions of weaning regimes and perform non-inversive measurement of the saturation level of oxygen in blood steam of patients under ventilator session [18]. Achieving continuous transmission for in-blood oxygen measurement and ventilating performance may precipitate patient-ventilator synchrony while quantifying positive end-expiratory pressure (PEEP) delivered to patient as demonstrated by a study [19]. Guler et al 2014 developed a self-intuitive computer-based interface to regulate ventilation parameters and respiratory puts in critical care use of clinical ventilators [20, 21]. There are raising innovations around computer-mediated interventions in the utilization of patient-interfacing ventilators for modeling may be preceded as a tool for improving the applicability of the ventilator device.

The review of work done by various authors show that many Mechanical Ventilator are not automated, they must be adjusted manually by the clinicians. Involvement of Clinicians is required to determine operating parameters of the device. This could translate to sources of operational error, which may limit the performance of the Ventilator as life support device. Therefore, there is a critical need for a complete automation of the electro-pneumatic systems, which could assist in improving patient support during the use of clinical ventilators.

In this research, an archetypal model of electropneumatic pathways for computer-controlled ventilation is developed, with dependable degree of patient-ventilator symphony over intermediate regimes of ventilating session. Pertinent parameters such as tidal volume, exhaust timing and exhalation timing sequence are determined. The performance of auto-delay mechanisms in the ventilating circuit is investigated while the observed implications on ventilating performance are explained in this study. A suitable solenoid valve was considered the best with consistent flowrate. This newly developed automated ventilator will overcome the operational errors often found in the existing ventilators.

## 2 Materials and method

The study considered an unsteady laminar, incompressible flow through the collapsible area of pneumatic ducts, which is a representative of the native upper respiratory tract. The prediction of the breathable air circuit is modelled by utilizing physical laws and established computational scheme subject to the assumptions that:

(i) The air in the system follows all ideal gas laws
(ii) The dynamic process is a quasi-balanced process
(iii) There is no air leakage during the working process

**Volume Equation**

Subject to the assumptions stated above, the output flow rate of a volume-controlled ventilator as described by [22] can be stated as:

(1) $${\text{Q}}_{\text{vo}}=\left\{ \begin{array}{ll} {\text{Q}}_{\text{T}}, & {\text{Q}}_{\text{vo}}<\;{\text{V}}_{\text{T}}\\ 0, & {\text{Q}}_{\text{vo}}\geq \;{\text{V}}_{\text{T}} \end{array}\right.$$

Where, ${\text{Q}}_{\text{T}}$ = $\frac{{\text{V}}_{\text{T}}}{{\text{T}}_{\text{vo}}}$ = $\frac{{\text{V}}_{\text{T}}}{{\text{T}}_{\text{i}}-{\text{T}}_{\text{p}}}$

The mass flow rate of breathable air can be modelled with Eq. (1) while the ventilating pressure range utilized is 0–40 cm H_2_O while the difference between the upstream and downstream pressure is ∼ > 0.528.

**Pressure Equation**

The flowrate in respect to the pressure within convective airways is represented by:

(2) $${\text{Q}}_{\text{ev}}=\frac{{\text{A}}_{\text{ev}}{\text{ ​p}}_{\text{t}}}{{\text{p}}_{\text{a}\sqrt{\text{θ}}}}\sqrt{\frac{2k}{k-1}\;\frac{1}{r}}\left[{\left(\frac{p_{l}}{p_{t}}\right)}^{2/k}-{\left(\frac{p_{a}}{p_{t}}\right)}^{\left(k+1\right)/k}\right]$$

(3) $${\text{Q}}_{\text{rt}}=\frac{{\text{A}}_{\text{rt}}{\text{ ​p}}_{\text{t}}}{{\text{p}}_{\text{a}\sqrt{\text{θ}}}}\sqrt{\frac{2k}{k-1}\;\frac{1}{R}}\left[{\left(\frac{p_{l}}{p_{t}}\right)}^{2/k}-{\left(\frac{p_{l}}{p_{t}}\right)}^{2/k}-{\left(\frac{p_{l}}{p_{t}}\right)}^{\left(k+1\right)/k}\right]\;p_{t}\;>p_{l}$$

Where ${\text{Q}}_{\text{ev}}$ is the Quick exhaust valve; ${\text{Q}}_{\text{rt}}$ is the mean pressure, and the compliance of the lung described by Zheng et al 2013 [23].

(4) $$C_{1}=\frac{dV_{1}}{dp_{l}}$$

Considering the ventilation process as thermodynamic system where ventilation work is taken as an isothermal process and expressed using the established Clapeyron system of differential equations.

(5) $$\frac{\text{dp}}{\text{dt}}=\frac{1}{\text{V}}\text{Rθq}-\;\frac{\text{mRθ}}{{\text{V}}^{2}}\frac{\text{dV}}{\text{dt}}$$

After transformation of (3), the pressure in the flexible tube and the ventilated lung is

$$\frac{\text{dpt}}{\text{dt}}=\;\frac{\text{R}{\text{qV}}_{\text{t}}}{{\text{V}}^{2}\text{t}+\text{CmRθ}}$$

(6) $$\frac{\text{dpt}}{\text{dt}}=\;\frac{\text{R}{\text{qV}}_{\text{l}}}{\;{\text{V}}^{2}\text{l}+\text{CmRθ}}$$

### 2.1 Validation study

**Figure 1:**
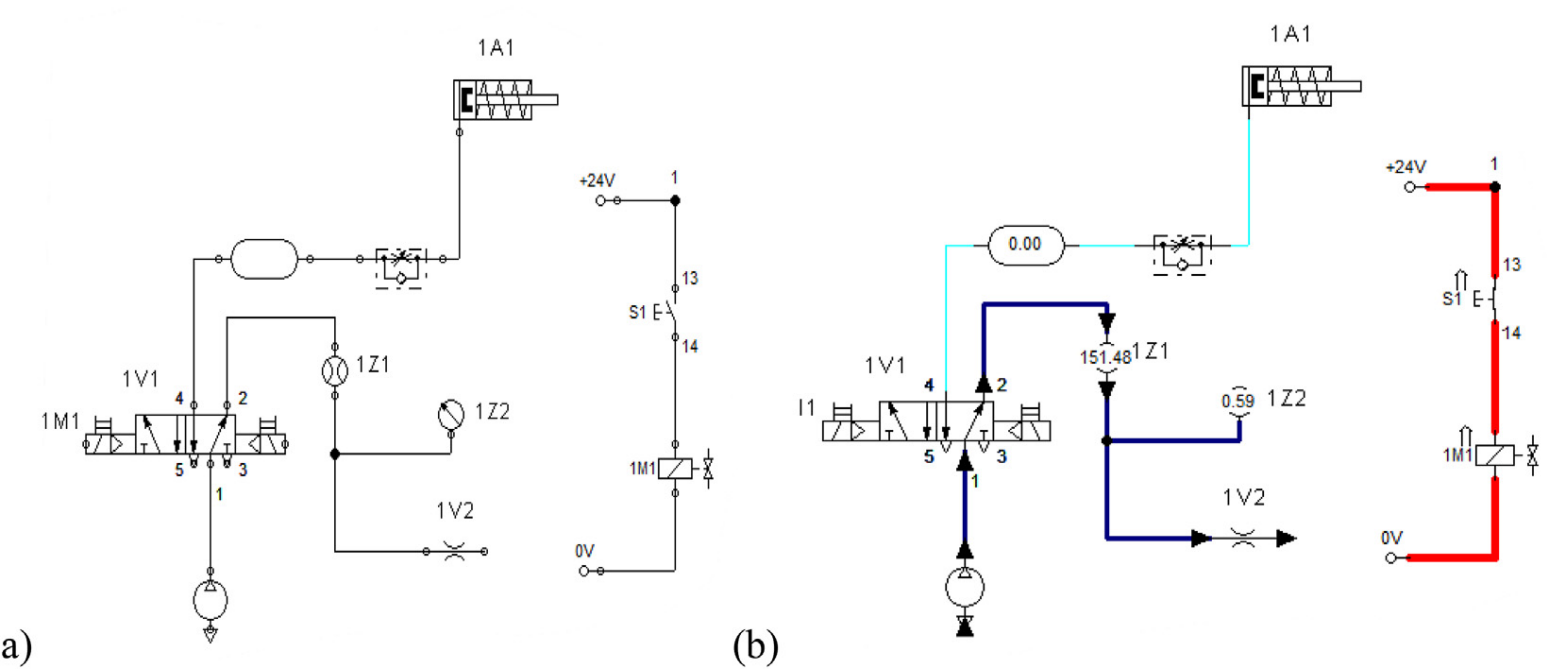
(a) Existing Model by Shi et al 2015, (b) Validation simulation structure diagram of the electro-pneumatic system for VCV on FluidSIM.

The air-current model of Shi et al 2014 is employed to validate the performance of solenoid-controlled pneumatic circuit developed in this study. The item of components similar to established report are implemented on computer-controlled pneumatic bench while modeling is complimented by using a fluidSIM software. The generation of pathways for breathable air is presented in Figure 1 and the parameters chosen for the validation are presented in Table 1.

**Table 1:** The parametric values setting of VCV for validation.

Effective Area $\left({\text{A}}_{\text{ev}}\right)$ of the Exhalation Valve. ${\text{mm}}^{2}$	Influence of the Tidal Volume. $\left({\text{V}}_{\text{T}}\right)$ mL	Influence of the breathable Time $\left({\text{V}}_{\text{vo}}\right)$ sec	Influence of the Tidal Pressure. $\left({\text{P}}_{\text{T}}\right)$ MPa
6	200	10	1
6	600	12	1.2
6	200	14	1.4
6	200	16	1.6

### 2.2 Experimental set-up for VCV

Table 2 shows the experimental design for Volume control Ventilator. PA denotes 5/2 solenoid valves, PB denotes 5/3 solenoid valves while PC denotes 3/2 solenoid valves. The time variants are denoted by 1, 2, 3 and 4 which represents 10 s, 12 s, 14 s and 16 s respectively.

**Table 2:** Experimental design for VCV.

S/NO	VCV				
1	PA	PA1	PA2	PA3	PA4
2	PB	PB1	PB2	PB3	PB4
3	PC	PC1	PC2	PC3	PC4

**Figure 2:**
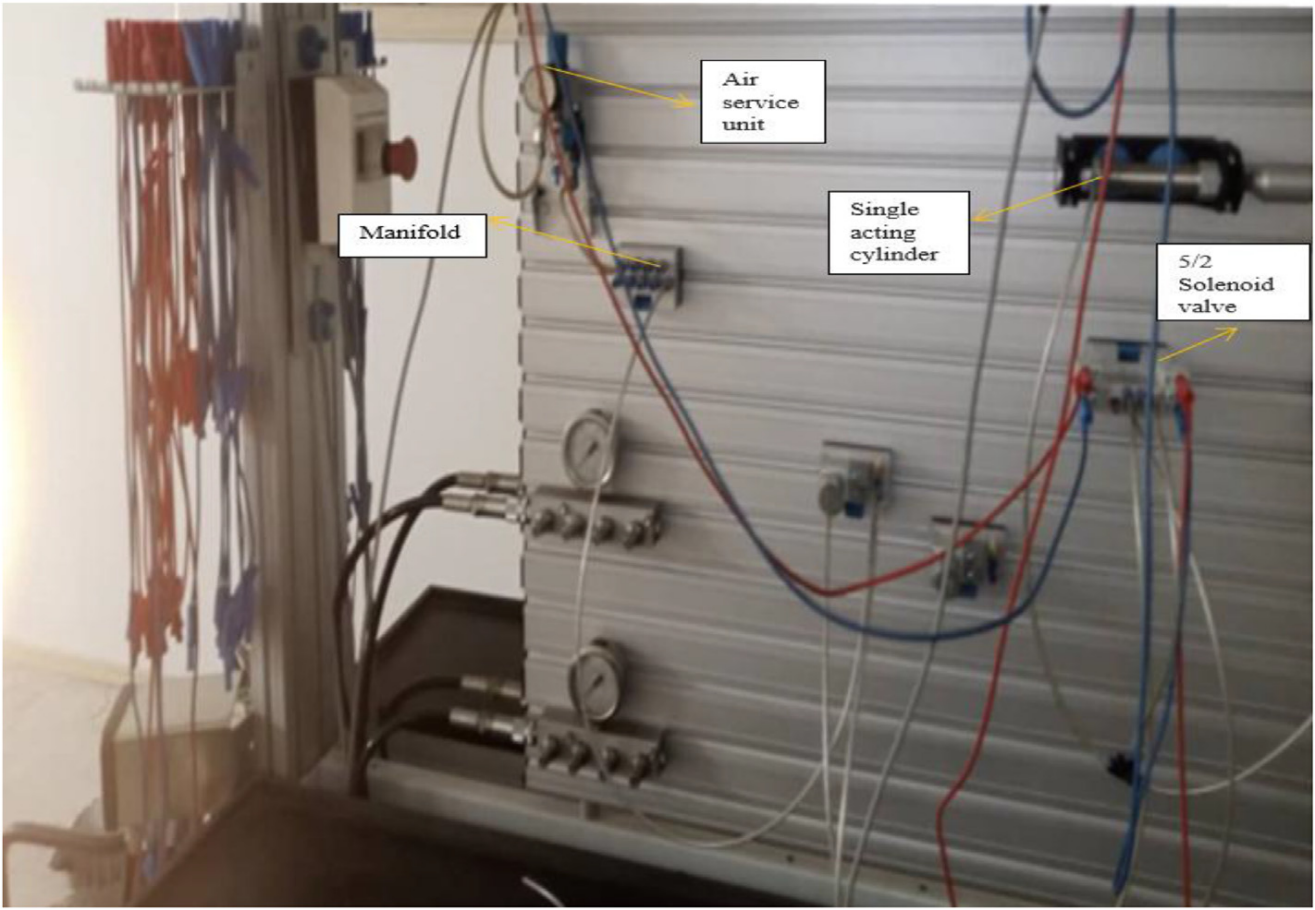
Experimental setup of EPCV for VCV.

The single-acting cylinder with detent cam and quick couplings is mounted on an advancing side, the unit is mounted on the profile plate using the quick release system with two nuts and three handles. The single-acting cylinder which has an attached trip cam and inbuilt push-in fitting, is mounted on a plastic retainer. This bitable 5/2-way solenoid valve with push-in fitting screwed to a function plate that is equipped with a port and silencer. The proximity sensor consists of the sensor of the mounting kit and cable, whereas the cable is equipped with a socket and three jack plugs as shown in Figure 2.

**Figure 3:**
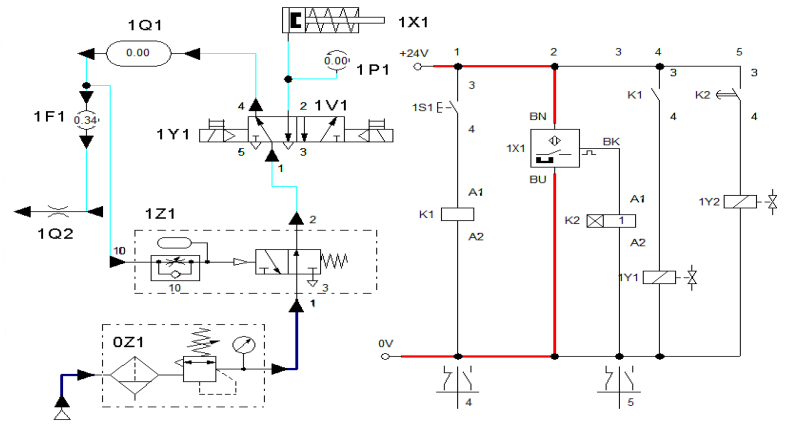
Electro-pneumatic simulation of circuit of VCV with 5/2 solenoid valve (PA).

If the piston is at the left stop in Figure 3, ports 1 (compressed air supply), port 2 (work port), port 4 (work port) as well as port 5 (exhaust port) are connected. If the left solenoid coil is energized, the piston moves to the right stop and ports 1 and 4 will be connected, while port 2 and 3 (exhaust port) will be connected. If the valve must return to the home position, it is not enough to interrupt current to the left solenoid coil, as the right solenoid coil must also be energized. If the solenoid could actuate the piston, maybe due to friction, then the piston will remain in its last assumed position (signal control in the power section). This also applies if the two solenoids are powered simultaneously, as they then act on each other with the same force, which in turn allows the valve to be switched by manual control in de-energized state.

In the initial position of the cylinder, 1 × 1 is in the forward end position and the rear cylinder chamber is filled with compressed air. A switching position remains the same as long as its actuator with a pushbutton. The function of a normally open contact is being performed by the pushbutton, in this case, the circuit is interrupted in the normal position of the pushbutton that is in the unactuated state. By actuation the switch button, the circuit become closed and subsequently allow the current to flow to the receiving device. Upon the release of the stem of the switch, the circuit is interrupted due to the immediate return of the pushbutton to the normal position being activated by the spring forces.

**Figure 4:**
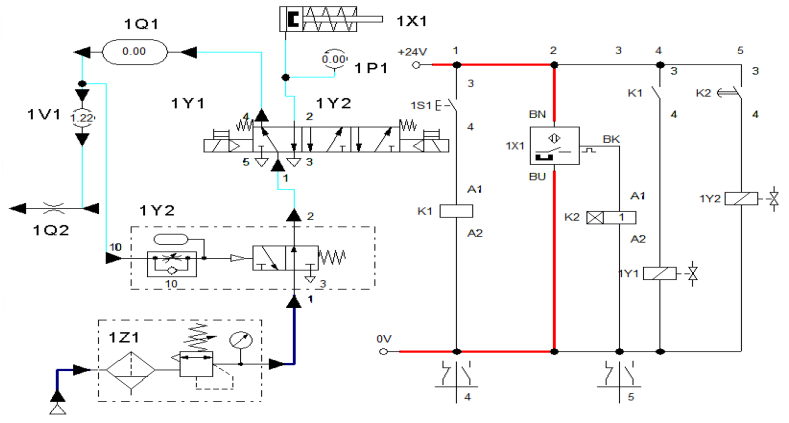
Electro-pneumatic simulation of circuit on VCV with 5/3 solenoid valve (PB).

Actuation of pushbutton 1S1 or pushbutton K1 (both in the form of normally open contacts), causes the relay K1 to be energized, the changeover contact K2 (connected in the form of a normally open contact) closes and the solenoid coil 1Y1 of the 5/3-way valve 1V1 is energized as shown in Figure 4. The valve 1V1 reverses and the rear chamber of cylinder 1 × 1 is exhausted; the spring presses the cylinder into the retracted end position. As soon as the pushbutton 1S1 or K1 (both in the form of normally open contacts), are no longer pressed, the relay K1 is de-energized, the changeover switch K2 (connected in the form of a normally open contact) opens. This causes the coil 1Y1 to be de-energize and the valve 1V1 to return into the initial position via the return spring. The rear chamber of cylinder 1Y1 is filled with compressed air and the cylinder returns into the forward end position.

**Figure 5:**
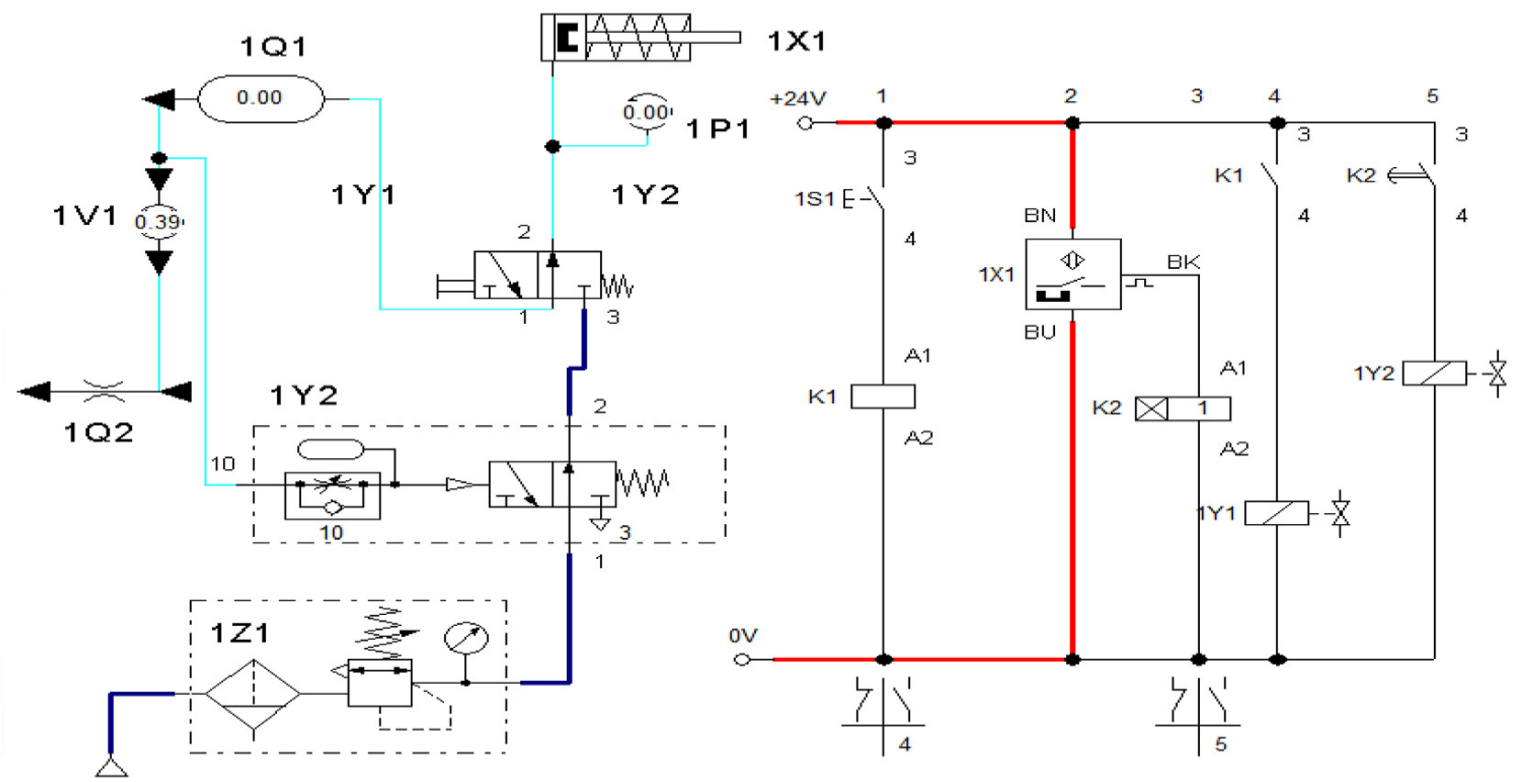
Electro-pneumatic simulation of circuit on VCV with 3/2 solenoid valve (PC).

This 3/2-way single solenoid valve with push-in fittings is attached to a function plate, which is equipped with a P port and silencer. The two electrical connections are equipped with safety connectors. The unit is mounted on the profile plate using a quick release detent system with a blue lever. The manifold with eight self-sealing push-in fittings is screwed on to a universal plate as shown in Figure 5.

## 3 Results and discussion

### 3.1 The result comparatives between the existing model and the new model for volume control ventilator

The data of the pressure in the flexible tube; the volume of the air flowing through the inlet of the flexible tube; the volume of the air entering and leaving the lung are shown in Table 3, which are compared with time in seconds. The key parameters of the VCV consist of its structural parameters and its settings such as Tidal volume and exhaust time. Using (PA) to represent 5/2 way solenoid valves, (PB) to represent 5/3 way solenoid valves and (PC) to represent 3/2 way solenoid valves in the table.

**Table 3:** Result of the existing Model (Shi et al 2014).

Volume (mL)	Pressure (MPa)	Flow rate (l/min)	Time (s)
300	1.2	6	1.0
500	1.6	8	1.2
600	2	12	1.4
700	2.5	12	1.6

The tidal volume of the ventilator is set to 300 ml, 500 ml, 600 ml and 700 ml, and the simulation results are shown in Table 4. It is found that with an increase in the tidal volume of the ventilator, the peak pressures in the existing model and the Electro-pneumatic VCV model increase proportionally, but when the tidal volume is greater than 600 mL, the maximum pressure in the existing model increases but slowly.

**Table 4:** Result of the Newly Developed model (Volume control ventilator) 5/2 valve.

Volume (mL)	Pressure (MPa)	Flow rate (l/min)	Time (s)
300	0.6	4.1	10
500	1	8.1	12
600	2	12	14.1
700	2.7	16.1	16

Influence of the evacuation time of the Ventilator. The exhaust time of the ventilator is set to 1 s, 1.2 s, 1.4 s, and 1.6 s, and the result of other study has been presented and validated in this study. Their result shows that the pressure platform in the pipe may not appear if the exhaust time of the ventilator is less than 1.6 s. on their model.

In addition, with an increase in the Electro-pneumatic exhaust time, the maximum pressure in the lung increases. When the exhaust time of the ventilator is more than 1.6 s, the maximum pressure in the lung is constant on Shi et al model. Finally, when the ventilator escape time is less than 1.6 s, the maximum lung flow increases with an increase in the discharge time. If the discharge time of the ventilator is greater than 1.6 s, the maximum flow rate is constant. The simulation results are consistent with the experimental results, which verify the mathematical model. Therefore, the mathematical model can be use in the study of VCV system, and it has better versatility and applicability. With an increase in the effective area of the exhalation valve, the maximum exhaust airflow rate of the lung.

### 3.2 Parametric investigation on VCV using programmable valve/standard actuator

The results of the three solenoid valves used in this study are presented in Table 5, in Electro-pneumatic control Ventilator, the peak point is faster than Shi et al model. At 1 s the curve begin to fluctuate, and we observe a stable curve at 10 s, which shows that 10 s, 12 s, 14 s and 16 s can be studied.

**Table 5:** Results of the three solenoid valves used for Volume Controlled Ventilator.

Volume (mL)	Pressure (MPa)	Time (s)	PA	PB	PC
			Flow Rate (l/min)	Flow Rate (l/min)	Flow Rate (l/min)
300	0.6	10	4.1	5	4.3
500	1	12	8.1	9	8.3
600	2	14	12	12	14.3
700	2.7	16	16.1	15.9	16

**Figure 6:**
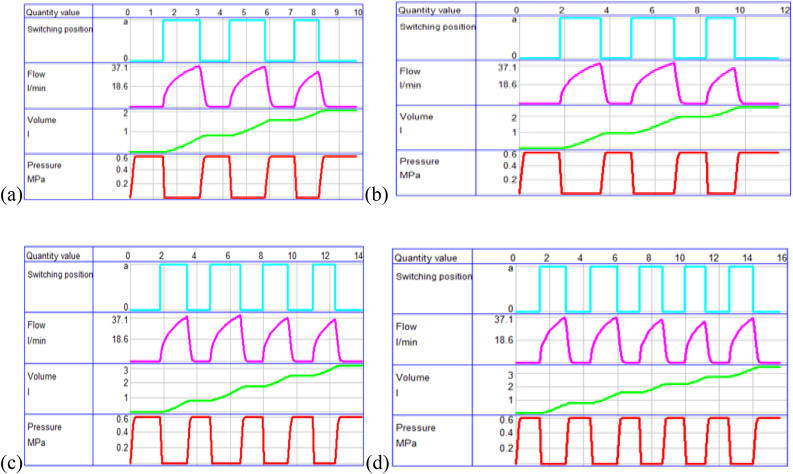
Simulation results of electro-pneumatic system of VCV using 5/2-way solenoid valves, (a) 10 s, (b) 12 s, (c) 14 s, (d) 16 s.

The experimental study of VCV on three different valves have been examined and simulation results of electro-pneumatic system of VCV in 10 s, 12 s, 14 s, and 16 s, using 5/2-way solenoid valves (PA) and single acting cylinder are shown in Figure 6.

**Figure 7:**
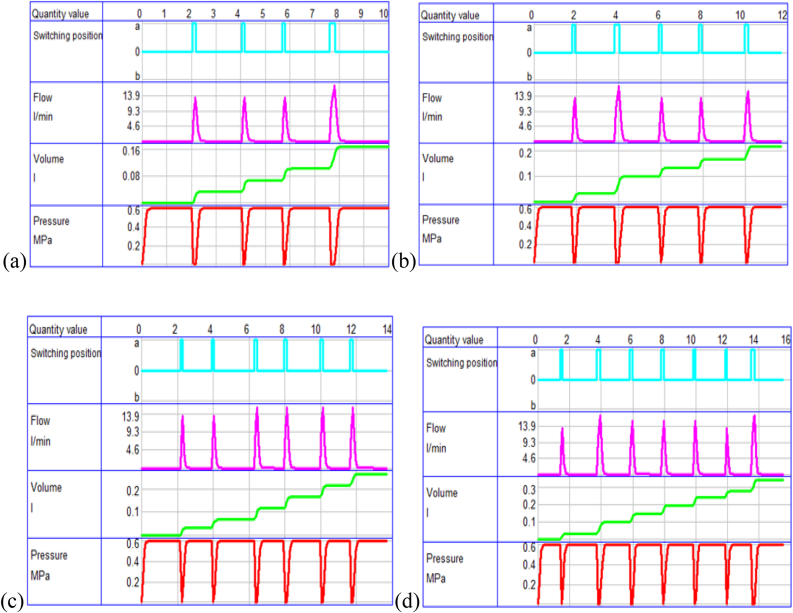
Simulation results of electro-pneumatic system of VCV using 5/3 way solenoid valves (PB), (a) 10 s, (b) 12 s, (c) 14 s, (d) 16 s.

For the 5/3 Solenoid valves, simulation results of electro-pneumatic system of VCV in 10 s, 12 s, 14 s, and 16 s, relay using 5/3-way solenoid valves (PB) and single acting cylinder. From the analysis of the actuator in Figure 7, the magnetic cushion of the single acting cylinder helps to reduce the sudden initial kick-off of the system from within the period of 0.2 s. This aids the slow down movement of the actuator in order to have good and stable exhaust without the application of an impulsive force, which could create a shock and a default on the lung.

**Figure 8:**
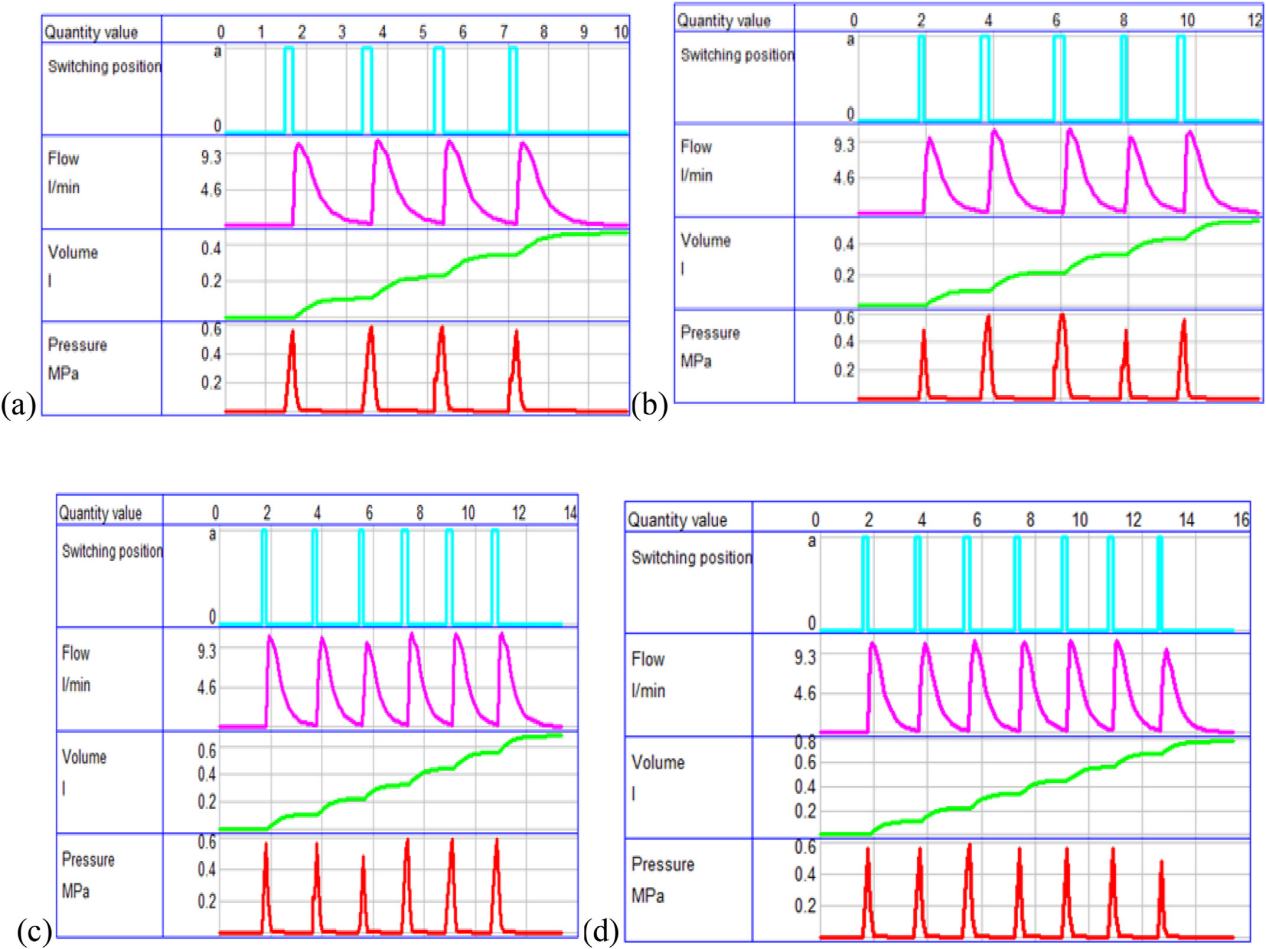
Simulation results of electro-pneumatic system of VCV in using 3/2 way solenoid valve, (a) 10 s, (b) 12 s, (c) 14 s, (d) 16 s.

Figure 8 shows the simulation results of VCV in 10 s, 12 s, 14 s and 16 s relay using 3/2 way solenoid valve (PC) and single acting cylinder. The linearity of tidal volume, peak pressure and lung compliance were observed for some selected parameters. The 5/2 solenoid valve can be seen as the best to be used, as it has a flowrate of 4.1 l/min for 10 s, 8.1 l/min for 12 s, 12 l/min for 14 s and 16.1 l/min for 16 s.

## 4 Conclusion

This study investigated the parametric modelling of clinic-bound mechanical ventilators of volume-controlled variant. The Clinical ventilator is relatively a system that employs several valves and sensors along with a processing unit to implement the required control algorithms. The performance of auto-delay mechanisms in the ventilating circuit is investigated while the observed implications on ventilating performance in terms of the linearity of tidal volume, the peak pressure and lung compliance were observed for some selected parameters. The 5/2 solenoid valve was considered the best with consistent flowrate of 4.1 l/min for 10 s, 8.1 l/min for 12 s, 12 l/min for 14 s and 16.1 l/min for 16 s. This newly developed automated ventilator will overcome the operational errors often found in the existing ventilators and It can be stated that the performance of VCV system in terms of peak time, settling time and maximum overshoot are automatic.

The relationship between tidal volume of breathable air and compliance of model lung of selected class of delay-time provides better results as compared to other models. The archetypal model of pneumatic circuit developed in this research could find vital application in the design of patient-interfacing devices particularly in ventilators and neonatal incubator.

## Declarations

### Author contribution statement

Ebenezer Olubunmi Ige: Conceived and designed the experiments; Analyzed and interpreted the data.

Adedotun Adetunla: Conceived and designed the experiments; Performed the experiments; Wrote the paper.

Samuel Olufemi Amudipe: Performed the experiments; Wrote the paper.

Adeyinka Adeoye, Matthew Glucksberg: Contributed reagents, materials, analysis tools or data.

### Funding statement

This research did not receive any specific grant from funding agencies in the public, commercial, or not-for-profit sectors.

### Data availability statement

The data that has been used is confidential.

### Declaration of interests statement

The authors declare no competing interests.

### Additional information

No additional information is available for this paper.
